# Rare genital malformations in women’s health research: sociodemographic, regional, and disease-related characteristics of patients with Mayer-Rokitansky-Küster-Hauser syndrome

**DOI:** 10.1186/s12905-020-00969-9

**Published:** 2020-06-29

**Authors:** Sara Yvonne Brucker, Leonie-Sophia Pösch, Joachim Graf, Alexander N. Sokolov, Norbert Schaeffeler, Andrea Kronenthaler, Hanna Hiltner, Anke Wagner, Esther Ueding, Monika A. Rieger, Dorit Schöller, Diana Stefanescu, Kristin Katharina Rall, Diethelm Wallwiener, Elisabeth Simoes

**Affiliations:** 1 University Hospital Tübingen, Department of Women’s Health, Tübingen, Germany; 2 University Hospital Tübingen, Department of Women’s Health, Research Institute for Women’s Health, Tübingen, Germany; 3 University Hospital Tübingen, Institute for Health Sciences, Section of Midwifery Science, Tübingen, Germany; 4 Department for Psychiatry and Psychotherapy, University Hospital Tübingen, Tübingen, Germany; 5 University Hospital Tübingen, Internal Medicine, Department of Psychosomatic Medicine and Psychotherapy, Tübingen, Germany; 6 University of Tübingen, Department of Sociology, Tübingen, Germany; 7 University Hospital Tübingen, Institute of Occupational and Social Medicine and Health Services Research, Tübingen, Germany; 8 University Hospital Tübingen, Staff Section Social Medicine, Tübingen, Germany

**Keywords:** Mayer-Rokitansky-Küster-Hauser syndrome MRKHS, Rare disease, Primary amenorrhea, Transition care, Health care research, Socio-demographics

## Abstract

**Background:**

The Mayer-Rokitansky-Küster-Hauser syndrome, MRKHS, is a rare (orphan) disease characterized by the aplasia or hypoplasia of the uterus and the vagina. In women's health research, little is known as to how much care provision for patients with MRKHS takes into account their socio-demographic together with their clinical characteristics. This work examines the patients’ socio-demographic characteristics, highlighting issues of inappropriate and deficient provision of care.

**Methods:**

The study was carried out as part of the larger TransCareO project and included a group of *N*=129 MRKHS patients who underwent surgery between 2008 and 2012. Using a specially developed questionnaire, we analyzed MRKHS patients’ data found both in the clinical documentation of the Department for Women's Health, University Hospital of Tübingen and the patient surveys of the Center for Rare Genital Malformations (CRGM/ ZSGF). Patients who took part in interviews were compared with non-respondents.

**Results:**

Patient respondents and non-respondents did not differ as to the parameters of interest. In most cases, primary amenorrhea was reported as an admission reason. In 24% of patients, a medical intervention (hymenal incision or hormone treatment) already occurred before admission to the Center in Tübingen and proper diagnosis of MRKHS. About one third received in advance inappropriate treatment. During the therapy, more than half of the patients were in a solid partnership. 10% of the family anamneses documented the occurrence of urogenital malformations.

**Conclusions:**

Care provision for MRKHS patients is largely characterized by delayed proper diagnosis and in part, by inappropriate treatment attempts; there are also indications of regional differences. Anamnestic clues such as an asymptomatic amenorrhea or renal abnormalities of unclear origin still fail to result early enough in referral to a center on the basis of suspected MRKHS diagnosis. Urogenital malformations in the family are more common in patients than in the general population. For patients, a wide range of burdens are associated with the diagnosis. Abnormalities compared to their female peers occur, for instance, in the partnership status: MRKHS patients have more rarely a partner.

## Background

### (Epidemiological) importance of the Mayer-Rokitansky-Küster-Hauser syndrome

The Mayer-Rokitansky-Küster-Hauser syndrome (MRKHS) is a malformation of the female genitalia with aplasia or hypoplasia of the uterus and the vagina as a result of an improper merger of the Müllerian (paramesonephric) ducts in the second month of embryo development [1–3]. The malformations vary in their extent [1–5]. One can distinguish MRKHS of Type I and Type II: Type I is diagnosed with a solely vaginal and uterine aplasia [4–7], while Type II (atypical form) is associated with other malformations, especially in the renal and skeletal system [4–15]. Type I patients are more common than patients with associated malformations [16], although data on their proportions vary in the literature [17]. In respect to etiology, a multifactorial pathogenesis is currently assumed [16, 18]. It is proposed that different chromosomal regions and gene structures could be linked to the emergence of MRKHS [18–21]. Clinically, MRKHS comes in appearance mostly due to missing menarche in puberty; the most common reasons for the first presentation at the gynecologist are the primary amenorrhea without pelvic pain and an anatomically conditional cohabitation inability [1, 22, 23]. MRKHS belongs to the rare (orphan) diseases with the annual incidence of 1:4,000 up to 5,000 of female live births [4, 24]. In 15 % of all patients with primary amenorrhea, this is caused by a MRKHS [25]. At the time of diagnosis, the affected women are in a phase of life that due to puberty is characterized by both physical and psychosocial changes and crises, and essentially shaped by the development of female identity and sexuality [26–29]. In this period of life with the transition from childhood to adulthood, the diagnosis of MRKHS presents a great emotional and mental burden for patients, in particular due to the sterility and the lack of cohabitation ability, since MRKHS affected patients cannot have sexual experiences unlike their peers. Accordingly, the increase of quality of life, including a possibility of cohabitation [30] is among the objectives of the therapy through vaginal dilation [31] or laparoscopic surgery for the formation of a neovagina [32–34]. As women affected by MRKHS do not differ phenotypically from their healthy female peers and clinical symptoms are often missing prior to puberty, an early diagnosis is generally difficult. It often takes months from the first visit at the gynecologist until the day of the proper diagnosis. From the perspective of health care research, therefore, delays in diagnosis and treatment odyssey are characteristic of the patients.

### Rare genital malformations in women's health research

In women's health research, it is important to know the ways in which health care for patients with this rare genital malformation is provided and where deficits in care provision exist [35, 36], targeting the effectiveness dimension in the context of health care research [37]. For patients with these diseases, especially during the transition, i.e. when passing from pediatric to adult centered medicine, there is a need for continuous medical and psychosocial support. And particularly in the case of rare diseases, tailored offers are presently lacking in the framework of transition care [38, 39]. The literature points to a deficient care provision and support, drawing on limited knowledge of the needs of the patients [40, 41]. This applies in particular to MRKHS patients [42, 43]. So far, it is also unknown if there are differences between more rural and more urban regions and to what extent the disease associated psychosocial stress affects the individual lives of the affected persons.

### Aims

In the framework of the overarching health care research project TransCareO (Development of a provisional model to improve transitional care for female adolescents with genital malformations as an example of orphan diseases, BMBF support code 01GY1125), MRKHS patients who previously underwent surgery at the Department for Women’s Health, University Hospital of Tübingen, completed qualitative interviews with respect to their experience of care provision during the treatment of MRKHS and their existing needs of care and support [43, 44]. As part of the project’s overall research objectives, the present work examined the clinical and sociodemographic characteristics of MRKHS patients, highlighting issues of inappropriate and deficient provision of care during the transition phase. The present study intended through the analysis of the patients’ socio-demographic characteristics, to draw conclusions on the quality of care of patients with MRKHS, to uncover any (including residential status related) aspects of the inappropriate care, and to determine and identify potential areas for improvement. Furthermore, it was examined how often female MRKHS patients had a positive family history of urogenital malformations compared to the general population.

## Methods

### Patient population and study design

Female patients with secured MRKHS diagnosis who presented themselves between 2008 and 2012 at the Department for Women’s Health, University Hospital Tübingen, were invited by a letter to participate in the TransCareO health care research project. All patients were included who received a consultation and underwent surgery in the Department. The present study was based on data analysis of both the patients who expressed willingness to complete interviews with TransCareO experts (respondents; *N*_Resp_=24) and those who did not responded to the invitation letter (non-respondents; *N*_Non-Resp_=105). As no significant differences occurred between respondents and non-respondents regarding the socio-demographic and clinical parameters of interest, this retrospective study evaluated a total MRKHS patient population of *N*=129 persons. The TransCareO study was approved in advance by the Ethics Committee at the University and the University Hospital of Tübingen (project number 422/2012B01). Within the framework of the TransCareO project, a separate survey was developed specifically for this comparative analysis. The anonymized socio-demographic, residential status, anamnestic and disease-related data were collected partly from the digitized patient records and doctor letters and partly from patient questionnaires (Additional file 1) developed at the Center of Rare Genital Malformations/ Zentrum für Seltene genitale Fehlbildungen der Frau (CRGM/ ZSGF) for a previous study approved by the same Ethics Committee; No. 28/2008BO1. The questionnaires comprised items on the origin, family and social history, and medical care involved. Questions about the partnership status represented a special feature of the patient questionnaire at the Center in Tübingen. Differing information was found on some items. In the event of an inconsistency between the medical records and the patient’s self-reports, information was used primarily from the digitized medical records. In some cases, no relevant information was found such as, for example, in patients born relatively long ago (missing data). For this reason, some results were based on a smaller study population. According to the adopted data protection guidelines, all records were collected and assessed in a pseudonymized way, not allowing trace back individual patients.

### Statistical Analysis

Descriptive statistical data processing (frequency analysis) was carried out using statistical packages MS Excel 2010 and IBM SPSS 21 in order to gauge descriptive characteristics of the collected data regarding the socio-demographics, disease history, co-morbid conditions, and family anamneses. Importantly, socioeconomic aspects (educational level and partnership status), as well as disease history (age at diagnosis, duration between the first symptoms and the definitive diagnosis MRKHS, (mis) diagnoses before referral to the Center in Tübingen) were also analyzed in relation to the patients’ residential status in order to reveal any potential regional variability. For that purpose, the patients were assigned to two groups on the basis of their postal codes. Group 1 represented patients from the rural environment (communities or small towns with a population of < 10,000 inhabitants) while group 2 gathered patients from the urban environment (≥ 10,000 inhabitants, that is, small towns with at least basic central function, medium towns, and large cities). The data were not normally distributed as revealed by the Shapiro-Wilk test prior to the actual data processing. Statistical differences therefore were assessed using either the *χ*^2^-test (for dichotomous variables) or the Wilcoxon test. A two-sided (non-directional) *p*-value of <0.05 was considered statistically significant (α = 0.05). The plots were produced in MS Excel.

## Results

### Socio-demographic characteristics

Table 1 shows the sociodemographic characteristics of the total patient population. The mean year of birth was 1990, with the oldest and youngest included patients born in 1961 and 1997, respectively. The mean age of the patient population was 22.46 years in 2014 (at the time of the invitation letter to enroll in the study). A quarter of the patients stated that they were still in school at the time of admission to therapy in the Department of Women's Health. Surgical intervention and follow-up took therefore place under challenging psychosocial conditions, since due to the length of the post-operative care (stretching for months by means of a phantom) [1, 31], the patients were only hardly able to conceal their illness from their classmates. Nearly half of the patients (46%) had a primary or secondary school certificate, with one quarter claiming to have the general higher education entrance qualification. 55% of the patients reported living in partnership at the time of admission to the therapy, while about 8% (n= 10) pointed out that they were living together with their partner. 28% reported they were living in the federal state of Baden-Wuerttemberg (= state where the University Hospital Tübingen is located), the others came mostly from the federal states of Bavaria, North Rhine-Westphalia, and Lower Saxony. The residential addresses of 36% were assigned to the rural area (village, municipality or small town with ≤ 10,000 inhabitants), while 64% lived in the urban environment. With focus on partnership status and educational level, there were no statistically significant differences between rural and urban environment (Table 2).

**Table 1:** Socio-demographic characteristics

Socio-demographic attribute	
Year of birth	
*Mean (Median) [Min; Max]*	1990 (1991) [1961; 1997]
Age at time of the invitation letter for enrollment (in years)	
*Mean (Median)*	22.46 (21)
*Standard deviation (range (Min; Max))*	6.07 (37 (15; 52)
Highest level of education at the time of therapy admission ^a^	
*No graduation/ still at school*	n=32 (25%)
*School finished without graduation*	n=0 (0%)
*Primary school leaving certificate*	n=17 (13%)
*Secondary school leaving certificate*	n=42 (33%)
*Advanced technical certificate*	n=4 (3%)
*High school leaving certificate (“Abitur”)*	n=34 (26%)
Partnership status at the time of therapy admission	
*No partnership*	n=58 (45%)
*Living in partnership (married/ unmarried)*	n=71 (55%)
Residence at the time of therapy admission ^b^	
*Rural environment*	n=46 (36%)
*Urban environment*	n=83 (64%)

^a^Difference: up to main school (38%) vs. high school leaving certificate (62%): *χ*^2^(1)= 6.970, *p*= 0.0083

^b^Difference: rural (36%) vs. urban setting (64%): *χ*^2^(1)= 9.269, *p*= 0.0023

**Table 2:** Regional specifics from aspects of health care, treatment, and psychosocial burden of disease

Variable	Rural environment	Urban environment	p-value
Year of birth			
*Mean (Median) [Min; Max]*	1990 (1991) [1961; 1996]	1990 (1991) [1965; 1997]	0.728
Partnership status at the time of therapy admission *[with partner]*	*n*=27 (59%)	*n*=44 (53%)	0.534
Highest level of education at the time of therapy admission			
*No graduation/ still at school*	*n*=8 (17%)	*n*=24 (29%)	0.147
*School finished without graduation*	*n*=0 (0%)	*n*=0 (0%)	-
*Primary school leaving certificate*	*n*=9 (20%)	*n*=8 (10%)	0.110
*Secondary school leaving certificate*	*n*=17 (37%)	*n*=25 (30%)	0.427
*Advanced technical certificate*	*n*=0 (0%)	*n*=4 (5%)	-
*High school leaving certificate ("Abitur")*	*n*=12 (26%)	*n*=21 (25%)	0.922
Medical intervention before MRKHS diagnosis (Multiple answers possible) ^a^			
*No intervention*	*n*=34 (74%)	*n*=53 (64%)	0.243
*Stretching by one’s own*	*n*=2 (4%)	*n*=9 (11%)	0,296
*Hymenal incision*	*n*=6 (13%)	*n*=10 (12%)	0,870
*Hormonal treatment*	*n*=3 (7%)	*n*=11 (13%)	0,239
*Other*	*n*=3 (7%)	*n*=5 (6%)	0,911
Suspected diagnosis of MRKHS at the time of initial presentation at gynecologist (n=116)	*n*=26 of 42 (62%)	*n*=42 of 73 (57%)	0.646
Age at diagnosis (in years)			
*Mean (Median)*	16.17 (16)	16.23 (16)	0.687
*Standard deviation (range (Min; Max))*	1.51 (7 (14; 21))	3.20 (30 (2; 32)	
Time elapsed between onset of first abnormalities and diagnosis (in months)			
*Mean (Median)*	8.96 (2 )	12.24 (2)	0.592
*Standard deviation (range (Min; Max))*	30.38 (200 (0; 200))	32.94 (200 (0; 200))	

^a^Difference no intervention vs. (multiple) various interventions: rural, *χ*^2^(1)= 9.322, *p*= 0.0023; urban, *χ*^2^(1)= 6.557, *p*= 0.0

### Disease Biography

All patients had visited the Department for Women's Health because of physical complaints; most of the cases were referred to the Tübingen Center by the treating gynecologists (84%). In most of the patients, the referral diagnosis was primary amenorrhea (n= 117, 91%). According to anamnesis, cyclic abdominal pain was found in 15% (n= 19) complaints, 11% reported an attempted cohabitation, which failed. The proportion of patients who received prior medical intervention *before* MRKHS diagnosis was of relevance: 12% of the patients reported that they had received a hymenal incision or had undergone a hormonal treatment to provoke menstrual bleeding as physician thought he detected an uterus. The proportion of patients with previous hormone treatment was somewhat higher in the urban environment, but the differences were not statistically significant. The proportion of patients who did not receive any external treatment before MRKHS diagnosis at the Department of Women's Health of the Tübingen University Hospital was 64%. A total of 75 patients (65%) reported however that the suspected diagnosis of MRKHS had already been made at the time of initial presentation at gynecologist due to the described complaints. The proportion of patients with correct suspected diagnosis was slightly higher in a rural setting, but the differences were not statistically significant. The median age at conclusive diagnosis MRKHS was 16 years, the average time passed between the first contact with a doctor (mostly a gynecologist, partly a pediatrician or a general practitioner) due to abnormalities and MRKHS diagnosis was 11 months with a wide range of 0 up to 200 months. On average, more than three years passed from the correct diagnosis to the operative intervention in Tübingen (Table 3). In the youngest patient, the first signs of irregularities were already found at the age of 2 years, which is why their parents consulted a pediatrician at that time. However, she was diagnosed with MRKHS 200 months later at the age of 18, and had undergone surgery 5 months later.

**Table 3:** Disease biography: complaints, of anamnesis and health care relevant aspects

Variable	Manifestation
Somatic complaints at the time of therapy admission (Multiple answers possible)	
*Primary amenorrhea*	*n*=117 (91%)
*Cyclic abdominal pain*	*n*=19 (15%)
*Failed cohabitation attempt*	*n*=14 (11%)
*No complaints*	*n*=9 (7%)
Medical intervention before MRKHS diagnosis (Multiple answers possible) ^a^	
*No intervention*	*n*=82 (64%)
*Stretching on one’s own*	*n*=11 (9%)
*Hymenal incision*	*n*=16 (12%)
*Hormonal treatment*	*n*=15 (12%)
*Other*	*n*=8 (6%)
Suspected diagnosis of MRKHS at the time of initial presentation at gynecologist (n=116)	*n*=68 (59%)
Age at diagnosis (in years)	
*Mean (Median)*	16.21 (16)
*Standard deviation (range (Min; Max))*	2.71 (21 (2; 23)
Time elapsed between onset of first abnormalities and diagnosis (in months)	
*Mean (Median)*	11.08 (2)
*Standard deviation (range (Min; Max))*	31.97 (200 (0; 200)
Time elapsed between diagnosis and surgery (in months)	
*Mean (Median)*	3.24 (1)
*Standard deviation (range (Min; Max))*	5.18 (30 (0; 30)

^a^Difference: no intervention vs. (multiple) various interventions: *χ*^2^(1)= 9.712, *p*= 0.0018

### Family history

Furthermore, the family anamnesis was analyzed to determine whether any other persons with MRKHS in the family or other genital or renal malformations were found in both female and male family members. In a total of 10% (n = 13) of the patients, a positive family anamnesis was found. Although this anamnesis was known, we found misdiagnosis of MRKHS also in this group. During the family anamnesis, the patients were asked about their known deformities in their parents and siblings. The proportion of patients with positive family history was significantly higher than in the general population.

## Discussion

### Essential results and limitations

The MRKH syndrome has increasingly become targeted by medical research in recent years due to particular attention to rare diseases and emerging therapeutic options such as uterine transplantation. Most studies on this rare genital malformation are primarily concerned with the causes, the variety of associated abnormalities [16, 45], and the establishment of new treatment options. The individual clinical care and treatment process as well as socio-demographic characteristics of the affected women receive, however, little attention. For this reason, the present study is of particular importance. Generally, the (operative) care for patients with rare diseases increasingly takes place at university centers [46], but diagnostic and care processes are usually tedious. For health care research and women's health research, it is of particular relevance, which clinical and socio-demographic characteristics are found in MRKHS patients and which areas are characterized by aspects of inappropriate health care. The aim of the present work was to identify any aspects of inappropriate health care for MRKHS patients – including those related to regional characteristics – in the context of socio-demographic variables and to point out potential ways for improvement. In total, n= 129 MRKHS patients were included in the analysis. More than half of the patients reported they had been in a partnership at the time of admission to the Department of Women's Health, and a quarter still had visited school. About one-third of the patients had previously received improper treatment; only 65% received MRKHS as accurate suspected diagnosis. Although 10% of the patients showed abnormalities in family anamnesis, including not only MRKHS but all known genital and associated malformations in both genders, misdiagnosis occurred also in this group. The relatively small sample size (typical of rare diseases) could be considered as one limiting factor. Particularly in the regional analysis, any differences found were indicative rather than statistically significant. Here, additional studies with the largest possible collectives are required in order to derive statistical significance for the non-significant differences indicated in the study. Mostly patients from southern and western Germany could be recruited to the study, while fewer patients from eastern and northern Germany participated. However, due to German history, differences may be expected especially for the East-West comparisons. The present results therefore could only with caution be considered representative of the German setting, requiring further research.

### Socio-demographic aspects

The mean age of the present population at final diagnosis was 16 years and was therefore within the transition age range. 55% of the patients reported that they were in a relationship at therapy admission and 8% lived together with their partners. In each case, the proportion was significantly lower than in the average female population of the same age group: in a German sample of 17-23 year old women, 89% of respondents reported they were in a solid or changing partnership (z= 12.342, p< 0.0001; 95% confidence interval, CI, of observed proportion, 46 to 63.77%) [47]. In the data report of the Federal Statistical Office, the proportion of 17- to 25-year-old women living together with their partner was 23% (z= 4.048, p= 0.0001; 95%-CI, 3.96 to 14.10%) [48]. These findings indicate that the malformations profoundly affect the partnership behavior and privacy of patients [49]. This implicates the existence of specific needs of the affected women ranging from the needs related to medium to long-term coping (such as family planning) to the need for psychotherapeutic support. As to the educational aspects, however, no much difference were found compared to the general population of the same age. As the average age of the study patients at diagnosis was 16.21 years, they were compared with the reference group of 15-20 year old women in Germany [50]. Here, further research is also needed as the lack of differences could result from the present sample size, while the outcome of qualitative interviews from the TransCareO project suggested substantial psychosocial stress potential particularly at school [43]. Other studies have shown that in adolescence, psychologically stressful situations can have a direct impact on academic performance [51], and school performance variability can be among the first concurrent psychosocial symptoms that should be better addressed, for example, through appropriate childcare provision at school [43]. An average of 11 months elapsed between the first abnormalities that led to the visit to the doctor and the diagnosis of MRKHS, which also highlights the need to stronger sensitize licensed gynecologists, general practitioners, and pediatricians for MRKHS and to enable them to promptly provide young women with such abnormalities with support measures such as communicating contacts to advisory services, specialist centers, psychotherapists or self-help groups. Study results suggest that initially, affected persons are interested in diagnostic safety (e.g., through a timely referral to a center) and then, in the second step either request support services or should be offered them as a cautionary measure [43]. The long period between the diagnosis and surgery (3 years on average) also provides evidence of insufficient psychosocial support, although it should be noted that not all patients want immediate surgery, but rather choose a more favorable time point because of the surgery`s complexity (e.g., between leaving high school and study begin at a university) [43]. This can also be seen from the fact that many patients indicated that they felt lost and insecure in the time until the final diagnosis was made. On average, patients preferred to have the invasive procedure in their 20th year of age, and thus usually after completing school education, on the transition to a new stage of life. During interviews with affected women in the context of the health care research project TransCareO, it became clear that this time period was often chosen intentionally for operative therapy in order to attract as little attention as possible and to avoid questions [43, 44, 52]. This would be a possible explanation for the timing of the surgical treatment, which is ultimately determined by many patients themselves after detailed consultation. Although differences between rural and urban environments were found in this sample, they did not attain statistical significance. Such differences, however, could be expected (e.g., in relation to duration of diagnosis in favor of the urban environment and level of psychosocial support in favor of the rural environment), and this should be investigated in future studies.

### Family history

**Fig. 1:**
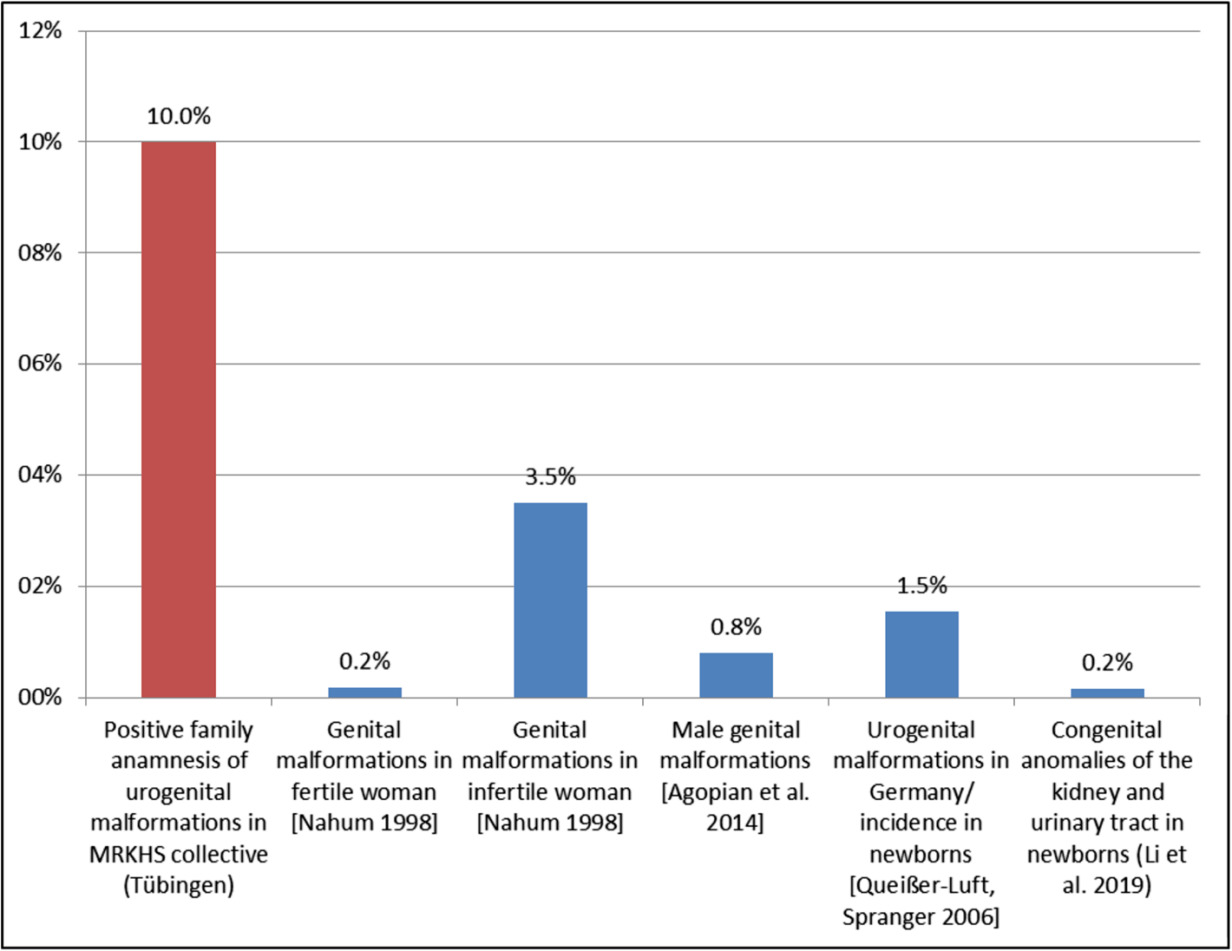
History of genital malformations in MRKHS collective and prevalence of genital malformations in the total population

In the case of asymptomatic amenorrhea and / or vaginal aplasia in combination with primary amenorrhea and / or genital or urological malformations in the family anamnesis, the treating gynecologists, general practitioners, and pediatricians should issue a referral to a specialized center on the basis of the differential diagnosis of MRKHS. The present study clearly shows that health care for MRKHS in patients of transition age is characterized by aspects of inappropriate care, which not only have a negative effect in patient-centered view, but also may result in unnecessary costs for the health care system itself. This is evident not only in the extended period of time between the initial contact with a doctor (due to abnormalities) and the ultimate MRKHS diagnosis. Thus, a quarter of the patients reported having received medical interventions (e.g., hymenal incision or hormone treatment) *before* the suspected diagnosis of MRKHS was made. One third of the patients had first been diagnosed in the Department of Women's Health in Tübingen, so patients with MRKHS obviously do not receive an appropriate care. This high proportion of cases points out to the need to anchor MRKHS as a rare genital malformation more strongly in medical training and further education. Advances can be made through intensified activities in this regard [40]. Finally, the fact that one out of every ten MRKHS patients in this sample had a positive family anamnesis of genital or renal malformations (since MRKHS Type II is often associated with other malformations, especially in the renal and skeletal system [4–15]), underscores the need for a thorough (social) anamnesis, especially in the gynecological setting, to identify patients with MRKHS, more quickly and more efficiently [53]. In the total female population, prevalence of genital malformations is assumed to be 0.2 (in fertile women) to 3.5% (in unfertile women )[54–56] and in men 0.8% [57], with estimated incidence of urogenital malformations in Germany of 43 to 154 per 10,000 live births (incidence in newborns: 0.43% - 1.54%) [58, 59], while the average prevalence of congenital anomalies of the kidney and urinary tract in newborns is around 1.60 per 1000 births (incidence rate: 0.2%) [60]. Thus, the prevalence of urogenital malformations in the study patients’ families (10%) was significantly higher than in the total population (z= 2.606, p< 0.01; 95%-CI, 5.42 to 16.52) (see also Fig. 1). The prevalence of urogenital malformations was significantly higher than the prevalence of genital malformations in the literature as well as that of renal malformations and cumulative values.

## Conclusions

In order to improve health care of patients with rare genital malformations (and specifically, MRKHS), the present work was carried out within the larger TransCareO project using a mixed-method design. Earlier findings revealed a high previously unknown need for information on disease and specialized health care in affected women [52]. For the first time in the international research context, the present work analyzed socio-demographic data in relation to the educational and the partnership status of the patients with MRKHS, targeting the psychosocial burden of disease. In this respect, further research and activity is required to implement target-group support services, whereby socio-demographic findings (particularly with regard to partnership status) indicate a high level of psychosocial burden.

## Supplementary information

**Additional file 1.** Short questionnaire on sociodemography in the context of the interviews.

## Abbreviations

*CI*: Confidence interval
*CRGM (ZSGF)*: Center for Rare Genital Malformations (Zentrum für Seltene Genitale Erkrankungen)
*e.g.*: exempli gratia, for example
*i.e.*: id est; that is to say
*MRKHS*: Mayer-Rokitansky-Küster-Hauser syndrome
*SPSS*: Statistical Package for the Social Sciences
*TransCareO*: Development of a provisional model to improve transitional care for female adolescents with genital malformations as an example of orphan diseases
