# Supplementary material for: Rare genital malformations in women’s health research: sociodemographic, regional, and disease-related characteristics of patients with Mayer-Rokitansky-Küster-Hauser syndrome
Source: BMC Womens Health. 2020 Jun 29;20:135. doi: 10.1186/s12905-020-00969-9 (PMC7322870; doi:10.1186/s12905-020-00969-9)
Supplement: Supplementary file 1 — Additional file 1. Short questionnaire on sociodemography in the context of the interviews. [file 12905_2020_969_MOESM1_ESM.docx]

**Short questionnaire on sociodemography in the context of the interviews**

Baseline survey in the context of the interviews for the study " Development of a provisional model to improve transitional care for female adolescents with genital malformations as an example of orphan diseases” - development of the basics and quality criteria

To be filled in by the interviewer:

*Group:*

❑ Affected persons

❑ Handler (e.g. physician)

❑ Relatives

❑ Partner

Other:

Dear study participant,

With the following questionnaire we would like to ask for some basic information before the interview begins. This information is important for describing the group of participants in the study. The selection options are based on the surveys of the Federal Statistical Office (Destatis). Please answer each question by ticking the appropriate answer option. This information is anonymous.

**Please state your gender**

❑ female ❑ male

**Please state the year of your birth**

Year __________

**What is your nationality?**

❑ German ❑ Other:

**Where were your parents and grandparents born?**

­­­­­­­­­­­­­­­­­­­____________________________________________________

**Please indicate your marital/ partnership status**

❑ no partnership/ single

❑ with partner, unmarried/ no registered partnership

❑ married/ registered partnership

❑ living separately from married partner/partner in a registered partnership

❑ divorced / civil partnership dissolved

❑ widow/ registered partner deceased

❑ other:

**Do you have children?**

❑ no

❑ yes, biological children

❑ yes, adopted children/ foster children

**What is your current living situation?**

❑ alone

❑ alone with child(ren)

❑ with partner

❑ with partner and child(ren)

❑ with parents

❑ in institutions (e.g. boarding school, assisted living, ...)

❑ other:

**What is your highest general school leaving certificate?**

❑ no degree, student

❑ leaving school without a secondary school leaving certificate (elementary school leaving certificate)

❑ secondary school certificate (elementary school certificate)

❑ secondary school leaving certificate (Mittlere Reife)

❑ polytechnic secondary school of the GDR with completion of the 8th or 9th class

❑ polytechnic secondary school of the GDR with completion of the 10th grade

❑ A-levels for universities of applied sciences, completion of a technical secondary school

❑ general or subject-related higher education entrance qualification / Abitur

❑ secondary school leaving certificate via second educational path

❑ a different school leaving certificate, which is:

**If you're looking for closure: What is the degree you are seeking?**

❑ elementary school certificate

❑ secondary school leaving certificate, secondary school leaving certificate, technical school leaving certificate

❑ A-levels for universities of applied sciences, completion of a technical secondary school

❑ Abitur, general or subject-related higher education entrance qualification

❑ a different school leaving certificate, which is:

**Which professional training qualification do you have?**

❑ still in vocational training (preparatory year, trainee, intern, student)

❑ pupil and attend a vocationally oriented secondary school, technical college or similar

❑ I do not have a vocational qualification and am not in vocational training

❑ vocational training (apprenticeship) completed

❑ vocational school education (vocational school, commercial school, preparatory service for the middle service in public administration) completed

❑ training completed at a technical school in the GDR

❑ completed training at a technical college, master craftsman's school, technical college, vocational or technical academy

❑ Bachelor's degree from (technical) university

❑ University of Applied Sciences degree (e.g. diploma, master's degree)

❑ Universitätsabschluss (z. B. Diplom, Magister, Staatsexamen, Master)

❑ PhD

❑ A different professional qualification, namely:

**What is your current employment status?**

*gainfully employed:*

❑ white-collar, blue-collar, civil servant ❑ full-time ❑ part-time

❑ self-employed ❑ full-time ❑ part-time

❑ partial retirement (regardless of whether it is in the work or release phase)

❑ marginally employed, 400-euro job, mini-job

❑ "one-Euro-Job" (when receiving unemployment benefit II)

❑ occasionally or irregularly employed

❑ in vocational training/teaching

❑ in retraining

❑ military service/civilian service

❑ voluntary social year

❑ maternity, parental or other leave

❑ other:

*Not employed*

❑ pupil, student who does not work for money❑ arbeitslos

❑ housewife/ -man

❑ early retirement

❑ pension (early, old-age, widows-)

❑ occupational/disability pension

❑ other:
